# Reduced Levels of the Antiaging Hormone Klotho are Associated With Increased Aortic Stiffness in Diabetic Kidney Disease

**DOI:** 10.1016/j.ekir.2023.04.021

**Published:** 2023-04-30

**Authors:** Nikolaos Fountoulakis, Paraskevi-Maria Psefteli, Giuseppe Maltese, Luigi Gnudi, Richard C. Siow, Janaka Karalliedde

**Affiliations:** 1 Unit for Metabolic Medicine, School of Cardiovascular and Metabolic Medicine and Sciences, Faculty of Life Sciences and Medicine, King’s British Heart Foundation Center of Research Excellence, King’s College London, London, UK

**Keywords:** arterial stiffness, diabetic kidney disease, soluble Klotho, type 2 diabetes mellitus

## Abstract

**Introduction:**

Aortic pulse wave velocity (Ao-PWV) predicts cardiovascular and kidney disease in type 2 diabetes (T2D). Klotho is a circulating antiaging hormone (sKlotho) with putative cardiorenal protective effects. The relationship between sKlotho and Ao-PWV in diabetic kidney disease (DKD) is unknown.

**Methods:**

In a cross-sectional cohort study, the correlation of sKlotho measured by a validated immunoassay, and Ao-PWV measured by applanation tonometry, was investigated in 172 participants with T2D and early stage DKD (all had estimated glomerular filtration rate [eGFR] >45 ml/min) on stable renin angiotensin system (RAS) inhibition. In cultured human aortic smooth muscle cells (HASMCs) stimulated with angiotensin II (AngII), the effects of recombinant human sKlotho pretreatment were assessed on intracellular calcium ([Ca^2+^]_i_) responses and expression of proteins associated with proosteogenic HASMC phenotypes.

**Results:**

Mean (range) age of the cohort was 61.3 years (40–82) and 65% were male. Mean (±SD) Ao-PWV was 11.4 (±2.3) m/s, eGFR 78.8 (±23.5) and median (interquartile range) sKlotho of 358.5 (194.2–706.3) pg/ml. In multivariable linear regression analyses, we observed a statistically significant inverse relationship between sKlotho and Ao-PWV, which was independent of clinical risk factors for cardiorenal disease. Pretreatment of cultured HASMC with sKlotho significantly attenuated AngII-stimulated [Ca^2+^]_i_ transients and reduced osteogenic collagen (Col1a2) expression.

**Conclusions:**

In individuals with T2D and early DKD, lower levels of sKlotho are associated with increased Ao-PWV. Taken together with the direct effect of sKlotho on mediators of aortic wall stiffness *in vitro*, these findings may explain the enhanced risk of cardiorenal disease in DKD.

## Graphical abstract

**Figure:**
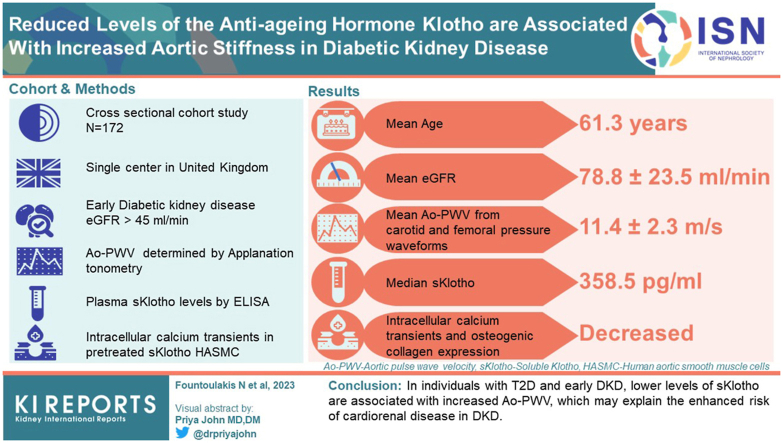

Aortic stiffness as estimated by aortic pulse wave velocity (Ao-PWV) is an independent predictor of cardiovascular morbidity and mortality.^1^ Enhanced risk for cardiovascular disease and kidney disease progression is present at early stages of DKD, which is also associated with accelerated vascular aging manifested by increases in Ao-PWV. Aortic smooth muscle cells play a key role in aortic stiffening^2^ and increased Ao-PWV is significantly correlated with calcification of the aortic intima and media.^3^ Osteochondrocytic transformation of vascular smooth muscle cells (VSMC) is the hallmark of vascular calcification.^4^ Imbalanced mobilization of [Ca^2+^]_i_ in response to prolonged AngII exposure is a key determinant of VSMC dedifferentiation that precedes osteochondrocytic transformation^5^ that is also underlined by extracellular matrix remodeling,^6^ partly via deposition of collagen.^7^

Klotho is a single-pass transmembrane protein mainly expressed in the distal convoluted tubule of the kidney where it serves as a coreceptor for the bone-derived phosphaturic hormone fibroblast growth factor (FGF)-23.^8^ A circulating, soluble form of klotho, sKlotho, quantifiable in plasma has been identified as an early biomarker of kidney disease and is an independent predictor of renal functional decline in people with diabetes.^9^^,^^10^ In the early stages of chronic kidney disease (CKD), there is an increased FGF-23-to-klotho ratio which is associated with vascular calcification. In a study by Yu *et al.*^11^ Klotho reduced hypertrophic growth of neonatal cardiomyocytes by inhibiting AngII-induced Wnt/b-catenin activation. Wnt/b-catenin can stimulate RAS gene expression leading to a decline in kidney function. It is hypothesized that increased FGF-23, Wnt activation, and the downregulation of sKlotho in CKD may promote volume overload and an elevation in blood pressure. However, the literature on the impact of AngII on vascular cell calcification is conflicting because a study has also described that^12^ the addition of Ang II decreased calcification in cells grown in a high phosphate medium by possibly reducing magnesium entry into cells.

A direct link between sKlotho deficiency and vascular calcification has been previously reported^13^; however, the relationship between arterial stiffness and sKlotho deficiency has not been fully elucidated in the context of DKD, which is characterized by accelerated vascular aging. In this study, we investigated the relationship between Ao-PWV and sKlotho levels in people with type 2 diabetes (T2D) and early stage DKD.

## Methods

### Clinical Characteristics

Participants were recruited from Guy’s and St Thomas’ Hospitals (London, UK) with the provision of informed written consent. The study was approved by the institutional research ethics committee and undertaken with adherence to the Declaration of Helsinki (Research study identifier number-10/H0802/86). All participants had T2D as defined by the World Health Organization criteria for diagnosis and classification of diabetes mellitus,^14^ were over 40 years of age and on stable doses of RAS inhibition. All participants had DKD with a history of microalbuminuria, defined by early morning urine albumin-to-creatinine ratio >3 mg/mmol on at least 2 occasions. Exclusion criteria included clinical or biochemical evidence of significant renal impairment (eGFR <45 ml/min) or history of non-DKD. There were no patients receiving vitamin D, phosphate binders, or sodium glucose transporter 2 inhibitors. This cohort included 91 participants’ data from baseline assessments (preintervention) from a previous study which investigated the effect of intervention with RAS inhibition or calcium channel blocker on Ao-PWV.

### Determination of Mean Arterial Pressure and Ao-PWV

Brachial blood pressure was measured in triplicate in the supine position by an automated sphygmomanometer (Omron Digital Blood Pressure Monitor HEM907, Bannockburn, IL). Mean arterial pressure (MAP) and pulse pressure (PP) were calculated from systolic blood pressure (SBP) and diastolic blood pressure (DBP) values according to the following formulas:

MAP = (SBP + 2 × DBP)/3 and PP = SBP − DBP.

Ao-PWV was determined from carotid and femoral pressure waveforms obtained noninvasively by applanation tonometry (Millar tonometer, Millar Instruments, Houston, TX) using the Sphygmocor system (Atcor, Sydney, Australia) as previously described.^15^ The distance between the surface markings of the sternal notch and the carotid (dc) and femoral artery (df) were used to estimate the path length between the carotid and femoral arteries (*L= df-dc*)) and Ao-PWV was computed as L/ΔT.^15^ The within-subject SD of Ao-PWV assessed by this method was 0.5 m/s in our laboratory. The intraobserver coefficient of variation was 3.5%.

### Biochemical Analyses

Plasma sKlotho levels were measured in duplicate by enzyme linked immunoassay (ELISA, Immuno-Biological-Laboratories, Hamburg, Germany).^11^^,^^16^^,^^17^ Blood samples were centrifuged immediately after collection at 1500 *g* (4 °C, 10 min) and the supernatants were stored at -80 °C (<24 months) with no freeze–thaw cycles before analysis. This approach has been shown to preserve the sensitivity of the immunoassay used herein.^16^ The intraassay and interassay coefficients of variation for sKlotho were 2.7% and 6.5%, respectively. Serum phosphate was measured in duplicate by spectrophotometry (Pointe Scientific Inc., Canton, MI).^11^ Serum total cholesterol (enzymatic colorimetry) and creatinine levels were measured using a Cobas Mira Plus analyzer (Roche Diagnostics, Rotkreuz, Switzerland),^18^ which was also used to determine urine albumin concentration by immunoturbidimetry from 3 timed overnight urine collections. The median albumin excretion rate (AER) was then calculated. glycosylated hemoglobin or hemoglobin A1c levels were detected by boronate affinity high performance liquid chromatography (Primus CLC330, Kansas City, MO). eGFR was determined using the Chronic Kidney Disease Epidemiology Collaboration Formula.^19^

### HASMC Culture and Treatment

HASMC (Lonza Group, Basel, Switzerland) were cultured in Dulbecco's Modified Eagle Medium (Sigma-Aldrich, St. Louis, MO) containing 10% (v/v) fetal calf serum, 1% (v/v) L-glutamine and penicillin/streptomycin (100 U ml^−1^/100 μg ml^−1^) in a 5% CO_2_/95% humidified air incubator at 37 °C as described before.^20^^,^^21^ Cells were passaged with trypsin and all experiments were performed between passages 6 and 10. Cells were treated with either recombinant human klotho (1 nM) (R&D systems, Abingdon, UK) or AngII (100 or 200 nΜ) (Sigma-Aldrich, St. Louis, MO), for the indicated periods of time.

### Intracellular Ca^2+^ Measurements

Intracellular [Ca^2+^] was monitored with the ratiometric fluorescent indicator Fura-2 AM (Invitrogen, Thermo Fisher Scientific, Waltham, MA) as described before.^22^ Briefly, HASMC cultured in clear-bottom black 96-well plates were pretreated with human recombinant sKlotho (1 nM, 24 h) before incubation with 2 μM of Fura-2 AM (45 min in dimethyl sulfoxide) at 37 °C. Subsequently, cells were equilibrated in Kreb’s buffer (mM: 131 NaCl, 5.6 potassium chloride, 25 sodium bicarbonate, 5 D-glucose, 1 monosodium phosphate, 0.1 L-arginine, 2 calcium chloride, and 1 magnesium chloride) for 30 minutes and baseline fluorescence was measured using a plate reader (CLARIOstar; BMG Labtech, Aylesbury, UK) at 340/380 nm excitation and 510/610 nm emission. AngII (100 nM) or vehicle (Kreb’s buffer alone) were then added using the integrated reagent injection system. The ratio of 340 out of 380 was used to calculate [Ca^2+^]_i_.

### Immunoblotting

HASMC were pretreated with sKlotho (1 nM, 24 h) before incubation with AngII (200 nM) for 72 hours in the continuous presence of klotho. Cell lysates were collected in a 2% w/v sodium dodecylsulfate buffer that contained protease inhibitors, and the amount of total protein was determined with the bicinchoninic acid assay (ThermoFisher Scientific, Northumberland, UK). Denatured samples were separated by SDS-PAGE, transferred to polyvinylidene difluoride membranes (Merck Millipore, Watford, UK), and probed with primary antibodies against α-actin (Santa Cruz Biotechnology, Dallas, TX), collagen type I alpha 2 (Col1a2) chain or matrix metalloproteinase-1 (Proteintech, Manchester, UK) and α-tubulin or β-actin (Millipore-Sigma, Burlington, MA) that were used as loading control proteins where appropriate. Protein expression was visualized with enhanced chemiluminescence and quantified by densitometric band analysis using FIJI software.^23^

### Statistical Analyses

Descriptive statistics were used for the analysis of demographic and clinical features of the cohort. AER and sKlotho levels were log-transformed for statistical analysis because of their skewed distribution. To assess whether sKlotho is independently associated with arterial stiffness, a multivariable linear regression analysis was performed with Ao-PWV as the dependent variable. In the multivariable model, we included variables associated with Ao-PWV at a *P*-value level of less than 0.1 in bivariate analysis as well as variables known to be associated with arterial stiffness in T2D and DKD.^24^^,^^25^ We used forced entry to include variables in the model and tested whether assumptions of linear regression were met. Statistical analyses were performed using SPSS software (version 26; SPSS Inc., Chicago, IL). We have constructed 2 separate multivariable models for variables associated with Ao-PWV using either MAP or SBP. Because SBP and MAP are biologically and clinically associated with and can influence Ao-PWV, we adjusted for each of these variables in our models. MAP incorporates both systolic and DBP and represents average blood pressure within a vessel over a contraction cycle of the heart muscle and hence influences Ao-PWV. Similarly, SBP is associated with and can determine Ao-PWV.^3^^,^^15^

For variables that had more than 5% up to 30% of missing data, we assumed that data were missing at random and used multiple imputation to complete missing values (mice package, R version 3.6.3). Statistical analyses for the clinical study were performed using SPSS software (version 26; SPSS Inc., Chicago, IL).

For studies using HASMC, data denote mean ± SEM of measurements in at least 3 independent HASMC cultures. A 2-way analysis of variance with Tukey multiple comparisons was used to compare the effects of sKlotho and AngII on [Ca^2+^]_i_, whereas the effect of sKlotho pretreatment on protein expression was evaluated by unpaired *t*-test. Graphical representation was performed with Prism (GraphPad Software, Inc, v7.0), also used to determine statistical significance at *P* < 0.05.

## Results

The baseline clinical and demographic characteristics of the 172 participants with T2D are shown in Table 1. The median (range) age of the cohort was 61.3 (40–82) years and 65% of the study population were male. Of the cohort, 42% were of White ethnicity, 46% of African-Caribbean ethnicity, and 12% of other ethnic background. Mean (±SD) eGFR was 78.8 (±23.5) ml/min with a median (interquartile range) AER of 36.6 (11.6–135.0) μg/min. Mean (±SD) Ao-PWV was 11.4 (±2.3) m/s and median (interquartile range) sKlotho levels were 358.5 (194.2–706.3) pg/ml.

**Table 1:** Demographic and baseline clinical characteristics of the study participants with type 2 diabetes and diabetic kidney disease

Cohort size	*N* = 172
Age, yrs	61.3 ± 9.1
Sex, % male	65
Ethnicity (White/Afro-Caribbean/Asian), %	42/46/12
Diabetes duration, yrs^a^	13.6 (2–34)
Receiving RAS inhibitors, %	100
Receiving insulin, %	37%
eGFR, ml min^−1^ 1.73m^−2^	78.8 ± 23.5
HbA_1c_, %	8.2 ± 1.6
HbA_1c_, mmol/mol	66.3 ± 17.5
Total cholesterol, mmol/l	4.3 ± 1.1
AER, mcg/min^b^	33.4 (13.6, 121.3)
SBP, mm Hg	152.0 ± 17.0
DBP, mm Hg	80.5 ± 9.9
MAP, mm Hg	104.3 ± 10.4
PP, mm Hg	71.6 ± 15.4
Ao-PWV	11.4±2.3
sKlotho, pg/ml^a^	358.5 (194.2, 706.3)

AER, albumin excretion rate; Ao-PWV, aortic pulse wave velocity; DBP, diastolic blood pressure; HbA1c, glycosylated hemoglobin or hemoglobin A1c; MAP, mean arterial pressure; PP, pulse pressure; RAS, renin angiotensin system; SBP, systolic blood pressure.

Data denote mean ± SD unless otherwise stated.

a Mean (range).

b Median (IQR).

The results of the bivariate association of various clinical and laboratory parameters with Ao-PWV are presented in Supplementary Table S1. There was no statistically significant correlation between eGFR and Ao-PWV (correlation coefficient −0.08, *P*-value = 0.3). There was modest statistical significance for the relation of MAP with Ao-PWV (correlation coefficient = 0.15, *P*-value = 0.046). Serum levels of calcium and phosphate do not correlate with Ao-PWV (correlation coefficients of −0.06 and −0.004 for calcium and phosphate respectively, *P* > 0.05). The association of eGFR with levels of sKlotho for the cohort is demonstrated in Supplementary Figure S1.

No statistically significant correlation of albuminuria (as measured by AER) with sKlotho (correlation coefficient of −0.03, *P*-value = 0.75) was observed in bivariate analysis.

There is no association between sKlotho and serum levels of calcium and phosphate (correlation coefficients of −0.005 and −0.017 for calcium and phosphate respectively, *P* > 0.05)

The results of 2 separate linear regression models, including adjustment for SBP (model 1) or MAP (model 2) as part of the predictor variables, respectively are presented in Table 2. Log-transformed sKlotho was an independent predictor of Ao-PWV in both models (β = −0.53, 95% confidence interval [CI] −0.96 to −0.10, *P* = 0.016 for model 1 and β = −0.69, 95% CI −1.11 to −0.27, *P* = 0.001 for model 2).

**Table 2:** Variables associated with Ao-PWV in 172 people with T2D and diabetic kidney disease

	Model 1			Model 2		
Variable	Beta coeff	95% CI	*P*-value	Beta coeff.	95% CI	*P*-value
Age	0.06	0.01 to 0.09	0.008	0.06	0.02 to 0.10	0.003
Gender	0.50	−0.21 to 1.21	0.17	0.58	−0.15 to 1.31	0.12
Ethnicity	−0.32	−0.81 to 0.18	0.21	−0.27	−0.78 to 0.23	0.30
eGFR	−0.008	−0.024 to 0.008	0.34	−0.008	−0.025 to 0.008	0.31
AER	−0.07	−0.28 to 0.13	0.48	−0.06	−0.27 to 0.15	0.6
SBP	0.029	0.007 to 0.05	0.009	N/A	N/A	N/A
MAP	N/A	N/A	N/A	0.014	−0.022 to 0.05	0.45
sKlotho	−0.53	−0.96 to −0.10	0.016	−0.69	−1.11 to −0.27	0.001

AER, albumin excretion rate; Ao-PWV, aortic pulse wave velocity; eGFR, estimated glomerular filtration rate; MAP, mean arterial pressure; SBP, systolic blood pressure.

Other variables included in the models were age (β = 0.06, 95% CI 0.01 to 0.09, *P* = 0.008 for model 1; and β = 0.06, 95% CI 0.02 to 0.01, *P* = 0.003 for model 2), gender (β = 0.5, 95% CI −0.21 to 1.21, *P* = 0.17 for model 1; and β = 0.58, 95% CI 0.15 to 1.31, *P* = 0.12 for model 2), ethnicity (β = −0.32, 95% CI −0.81 to 0.18, *P* = 0.21 for model 1; and β = −0.27, 95% CI −0.78 to 0.23, *P* = 0.30 for model 2), eGFR (β = −0.008, 95% CI −0.024 to 0.008, *P* = 0.34 for model 1; and β = −0.008, 95% CI −0.025 to 0.008, *P* = 0.31 for model 2) and log-transformed AER (β = −0.07, 95% CI −0.28 to 0.13, *P* = 0.48 for model 1; and β= −0.06, 95% CI −0.27 to 0.15, *P* = 0.6 for model 2). SBP (β = 0.029, 95% CI 0.007 to 0.05, *P* = 0.009) but not MAP (β = 0.014, 95% CI −0.022 to 0.05, *P* = 0.45) significantly predicted Ao-PWV.

The standardized beta coefficients of the multivariable model demonstrate a similar significant effect of age and SBP on Ao-PWV as sKlotho (standardized beta coefficients 0.21,0.22,0.20 for age, SBP and sKlotho, respectively)

**Figure 1:**
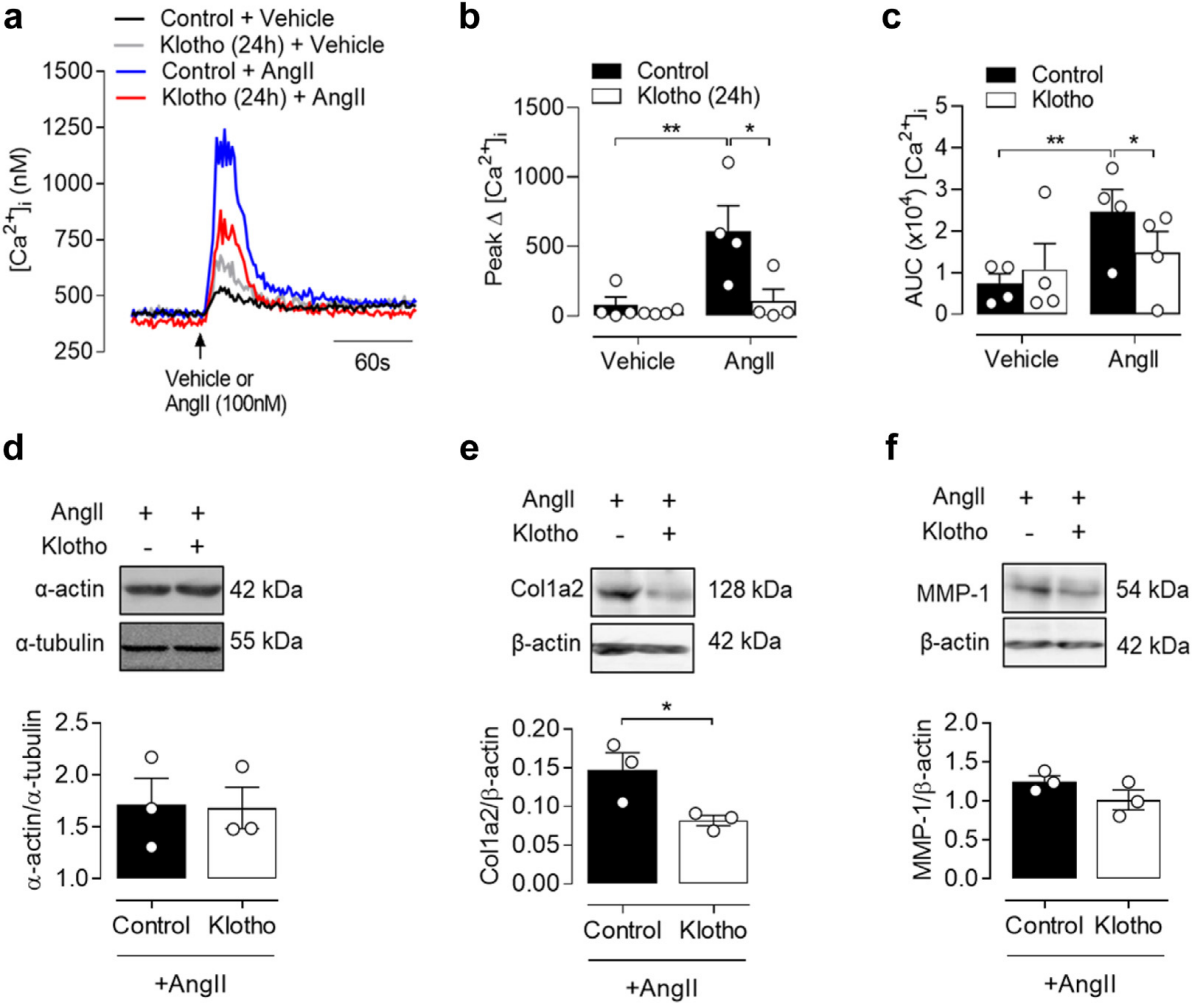
Soluble klotho attenuates AngII-stimulated [Ca^2+^]_i_ transients and reduces Col1a2 expression in HASMC. (a–c) Cells were treated with soluble klotho (1 nM, 24 h) before loading with the fluorescent Ca^2+^ indicator Fura-2 AM (2 μM, 45 min). [Ca^2+^]_i_ was monitored after stimulation (arrow) with AngII (100 nM) or Kreb’s buffer alone (Vehicle). (a) Mean [Ca^2+^]_i_ response and (b, c) mean ± SEM, baseline difference (Δ) from peak [Ca^2+^]_i_ (b) and area under curve (c) of [Ca^2+^]_i_ transients shown in (a) from 4 independent HASMC cultures. ∗*P* < 0.05, ∗∗*P* < 0.01, 2-way ANOVA. (d–f) HASMC were pretreated with soluble klotho (1 nM, 24 h) before exposure to AngII (200 nM, 72 h) in the continuous presence of klotho. Representative immunoblots and densitometric analyses of whole cell α-actin (d), Col1a2 (e) and MMP-1 (f) are shown relative to α-tubulin or β-actin loading controls. (d–f) Data denote mean ± SEM, from 3 independent HASMC cultures. ∗*P* < 0.05, unpaired *t*-test (2-tailed). Col1a2, collagen type I alpha 2; MMP-1, matrix metalloproteinase-1

The results of the multivariable models after inclusion of body mass index, glycosylated hemoglobin or hemoglobin A1c, serum calcium, and phosphate are presented in Supplementary Table S2. sKlotho remains significantly associated with Ao-PWV (β = −0.53, 95% CI −0.96 to −0.10, *P* = 0.016; and β = −0.69, 95% CI −1.11 to −0.27, *P* = 0.001 for the models for SBP and MAP, respectively). This study further investigated the molecular mechanisms through which sKlotho influences VSMC phenotypes associated with arterial stiffness. [Ca^2+^]i mobilization in response to AngII (100 nM) exhibited a typical biphasic pattern of initial rise and subsequent return to baseline with a small but sustained plateau phase (Figure 1a). HASMC pretreated with recombinant human klotho (24 h) exhibited a transient AngII-mediated [Ca2+]i increase, which was significantly reduced compared to control cells as indicated by the attenuated peak Δ[Ca^2+^]i (Figure 1a and b). Similarly, the initial rise in [Ca^2+^]i persisted less in sKlotho pretreated compared to control HASMC as indicated by the significant decrease in the area under the curve of [Ca^2+^]i responses (Figure 1c). In Figure 1, changes in [Ca2+]i transients of HASMC pretreated with sKlotho and acutely stimulated with AngII were measured from 4 independent HASMC cultures (please see figure legend). Figure 1a represents the mean raw trace of [Ca2+]i transients from the 4 experiments whereas the measurements from each individual experiment are represented by the white circles superimposed on the mean with SEM of each trace shown in Figure 1b and Figure 1c. For reference, a mean trace of [Ca2+]i from the same 4 experiments is included as a Supplementary Figure S2 and expressed as a fold-change from the baseline to facilitate direct comparisons. A reproducible injection artifact was expected during the real-time Fura-2 AM fluorescence measurements in all experimental conditions to be related with the injection of extra volume per well of a 96-well plate (equal volumes per condition) of either vehicle (Kreb’s buffer alone) or AngII (100 nM in Kreb’s buffer) using the integrated reagent injection system of the plate reader (CLARIOstar; BMG Labtech, Aylesbury, UK). Statistical comparisons presented in Figure 1b and c did not show significant differences between the peak Δ[Ca2+]i or area under the curve [Ca2+]i between the sKlotho+Vehicle and control+Vehicle conditions, respectively.

Next, given the functional role of dedifferentiated VSMC in vascular stiffening, the effects of sKlotho were evaluated in the context of long-term phenotypic HASMC changes. Protein expression was assessed in control or sKlotho pretreated cells exposed to AngII for 72 hours which, as we previously demonstrated, promotes HASMC senescence. Although sKlotho pretreatment did not alter α-actin expression (Figure 1d), it significantly reduced protein levels of Col1a2 (Figure 1e) and had a similar, although not statistically significant, effect on reducing the expression of matrix metalloproteinase-1 (Figure 1f).

## Discussion

The present study is the first to identify an inverse association between the circulating levels of the antiaging hormone klotho and aortic stiffness in people with T2D and early stage DKD. Moreover, we demonstrated that primary HASMC pretreatment with sKlotho significantly reduces AngII-stimulated [Ca^2+^]i transients and Col1a2 expression, both aspects of vascular smooth muscle phenotypes associated with aortic stiffness.

There are limited and conflicting studies examining the association between arterial stiffness and sKlotho, all of which predominantly include participants without diabetes. In a study of 114 patients with CKD stages 1 to 4, of which 11% had DKD, lower serum klotho levels were independently associated with higher brachial ankle pulse wave velocity.^26^ Kim and colleagues,^27^ showed a significant univariate association of sKlotho levels with several indices of pulse wave velocity including Ao-PWV in Korean patients with CKD; however, this association was not significant in multivariable analyses. A more recent community study in the general Chinese population also observed no significant association between Ao-PWV and sKlotho levels; however, less than 15% of the cohort had diabetes.^27^ In a study comparing patients with advanced DKD (majority) with healthy individuals,^28^ higher Ao-PWV and lower sKlotho levels were observed in DKD; however, no significant association between sKlotho levels and arterial stiffness was observed.^28^

There are several important differences between studies reporting no association between sKlotho and aortic stiffness and the present work. Most of previous studies report findings from participants without diabetes who, predominantly, did not receive RAS inhibitors. Furthermore, these studies included people with a wide range of kidney function levels. Diabetes *per se*, more advanced kidney disease (CKD stage 4 and 5) and RAS inhibition are all known to influence arterial aging as well sKlotho levels and Ao-PWV.^11^^,^^12^^,^^18^^,^^29^ In the study by Kim *et al.* mentioned previously, 33.9% of the participants had diabetes whereas 85.6% were on treatment with RAS inhibitors. Moreover, the authors looked across all stages of CKD, which was not exclusively caused by diabetes. Although there was statistical adjustment for the presence of DM and the use of RAS inhibitors in their model, the authors do not report the prevalence of DKD in their cohort and whether these patients were on standard of care treatment with RAS inhibition. In contrast, in our study, we excluded people with kidney disease not related to diabetes and chose a cohort with relatively preserved eGFR with eGFR >45 ml/min. Of note, advanced CKD, diabetes, and use of RAS inhibition will all influence both klotho and Ao-PWV or arterial stiffness.^30^

The conflicting data in this area of research is most likely related to the different inclusion criteria in different studies and the populations studied described above. Therefore, conversely to previous studies, we studied patients with T2D and DKD, who were all on RAS inhibition with eGFR above 45 ml/min. Moreover, we utilized Ao-PWV, the gold standard measure of aortic stiffness in contrast to several of the other studies which used brachial ankle or other indices of arterial stiffness.

There is no defined consensus or standardization of normal ranges for sKlotho levels with concentrations ranged from 239 to 1266 pg/ml (mean 562 ± standard deviation of 146 pg/ml) reported in healthy adults^31^ with levels known to decrease with decline in eGFR and progression to a higher stages of CKD.^30^, ^32^

Similarly for Ao-PWV there is no agreed or standardizes normal ranges defined with a rise observed with increasing age in healthy people. In a large population based study Ao-PWV values in people without cardiovascular disease, hypertension or diabetes ranged from a mean of 6.2 m/s in those under the age of 30 to 10.9 m/s in those above 70 years of age.^33^

In the 2018 European Society of Cardiology/European Society of Hypertension guidelines on the management of arterial hypertension a Ao-PWV > 10 m/s is considered an estimate of significant alterations in aortic function in middle aged hypertensive patients. Both diabetes and CKD accelerated vascular aging and increase Ao-PWV.^34^

In our cohort in people with diabetes and early stage CKD the dispersion of sKlotho and Ao-PWV values around a median and a mean of 358.5 pg/ml and 11.4 m/s respectively are expected findings although we acknowledge that especially for sKlotho there are no established and validated reference ranges either in healthy subjects or disease states such as diabetes and CKD.

We have previously demonstrated the protective effects of sKlotho against AngII-mediated oxidative stress, apoptosis, and senescence of HASMC.^20^ In this study, HASMC pretreatment with recombinant klotho did not affect alpha-actin expression but reduced both the magnitude and duration of AngII-stimulated [Ca^2+^]_i_ transients. The culture milieu utilized here was informed by the lack of significant differences in serum phosphate or calcium levels in our clinical cohort (data not shown). By culturing and treating HASMC in standard media, we aimed to recapitulate the early stages of T2D when renal function is preserved and before perturbations in mineral metabolism linked to kidney damage occur. This may explain why alpha-actin expression was not affected by sKlotho pretreatment. sKlotho was previously shown to preserve VSMC contractile function;, however, in the presence of high ambient phosphate levels.^35^ Moreover, the positive association between upregulation of RAS with large arterial remodeling is well-described in diabetes,^36^^,^^37^ an event paralleled by upregulation of vascular AngII type I receptor expression leading to vascular wall remodeling.^38^ Notably, AngII-mediated VSMC hypertrophy and hyperplasia are underlined by [Ca^2+^]_i_ imbalance,^5^ and sustained [Ca^2+^]_i_ increases are partly mediated by prolonged activation of AngII type I receptor.^39^ sKlotho can reduce cardiomyocyte hypertrophy via attenuation of AngII type I receptor signalling^8^ and via modulation of the expression and cellular localization of Ca^2+^-permeable channels such as TRPV5 and TRPC6 that regulate intracellular Ca^2+^ dynamics.^40^^,^^41^ sKlotho may therefore protect VSMC by ameliorating sustained [Ca^2+^]_i_ increases in response to AngII.

sKlotho pretreatment before chronic HASMC exposure to AngII also reduced Col1a2 protein levels, a key mediator of VSMC osteochondrocytic transformation. Our results are consistent with findings in cultured mouse aortic smooth muscle cells, where klotho gene deficiency increased autophagy, leading to upregulation of scleraxis, a key transcription factor of collagen synthesis.^42^ Similarly, in an animal model of klotho deficiency, increased Ao-PWV was associated with increased collagen expression and enhanced elastin fragmentation in the aortic media.^43^ Both extracellular matrix remodeling and VSMC migration in the arterial wall led to decreased vascular distensibility and involve matrix metalloproteinase-1 activity,^44^ which was unchanged in response to sKlotho pretreatment in our study. Reduced Col1a2 synthesis rather than increased degradation could therefore represent the protective mechanism by which sKlotho prevents the VSMC phenotypic switching that precedes vascular remodeling and causes arterial distensibility.

Our study has several limitations. Because of the cross-sectional study design, a causal relationship between lower sKlotho levels and higher Ao-PWV cannot be inferred. The number of participants studied was relatively small; however, this was in the context of selective inclusion criteria, which allowed us to only study patients with DKD and preserved renal function. We did not observe, in bivariate analyses, a significant association between Ao-PWV and FGF-23 and vitamin D in a subset of our cohort. However, a limitation of our study is that we did not have these measurements for the whole cohort and therefore further studies are needed to determine if these variables can influence the interrelationship between sKlotho and Ao-PWV. Because we excluded people with advanced CKD, which is more often associated with dysregulated FGF-23 levels and because we also excluded people receiving vitamin D or calcium or phosphate supplements it is unlikely these variables may have influenced our results, but further studies are needed to confirm this.

We also acknowledge the need for further experiments with HASMC in the presence of hyperglycemia. In T2D, hyperglycemia is one of multiple factors affecting vascular calcification and its impact also needs to be balanced against the effects of hyperinsulinemia.

In conclusion, people with T2D and increased Ao-PWV have lower circulating sKlotho levels and this may in part explain their enhanced risk of cardiorenal disease. Our findings in primary HASMC, where pretreatment with klotho negates the detrimental effect of RAS-mediated vascular changes offer a potential mechanism underlying our clinical observations.

The present findings establish the platform and scientific rationale for further research to elucidate the role of sKlotho on aortic stiffening and its potential use as a treatment to ameliorate aortic aging (reduce Ao-PWV) in diabetes and early stage renal dysfunction.

## Disclosure

All authors declared no competing interests.

## Supplementary Material

Supplementary File (PDF)

**Figure S1.** Association of sKlotho with eGFR in 172 people with T2D and DKD.

**Figure S2.** Soluble klotho attenuates AngII-stimulated [Ca2+]i transients.

**Table S1.** Bivariate correlation of PWV with various clinical and biochemical variables in 172 people with T2D and DKD.

**Table S2.** Variables associated with Ao-PWV in 172 people with T2D and diabetic kidney disease.
