# Supplementary material for: Reduced Levels of the Antiaging Hormone Klotho are Associated With Increased Aortic Stiffness in Diabetic Kidney Disease
Source: Kidney Int Rep. 2023 Apr 30;8(7):1380–8. doi: 10.1016/j.ekir.2023.04.021 (PMC10334399; doi:10.1016/j.ekir.2023.04.021)

**Supplementary Table S1. Bivariate correlation of PWV with various clinical and biochemical variables in 172 people with T2D and DKD.**		
	Pearson's correlation coefficient	p-value
**Klotho**	**-0.18**	**0.018**
FGF-23	0.14	0.15
Vitamin D	-0.15	0.32
eGFR	-0.08	0.3
**SBP**	**0.34**	**<0.001**
**BMI**	**0.03**	**0.69**
**MAP**	**0.15**	**0.046**
HbA1c	-0.06	0.44
**Age**	**0.16**	**0.03**
AER	-0.03	0.75
Calcium	-0.06	0.46
Phosphate	-0.004	0.96
FGF-23, fibroblast growth factor 23; eGFR, estimated glomerular filtration rate; SBP, systolic blood pressure; BMI, body mass index; MAP, mean arterial pressure; AER,albumin excretion rate		

**Supplementary Table S2. Variables associated with Ao-PWV in 172 people with T2D and diabetic kidney disease .**						
	Model 3			Model 4		
Variable	Beta coeff	95%CI	p-value	Beta coeff	95%CI	p-value
**Age**	**0.06**	**0.006 to 0.1**	**0.03**	**0.07**	**0.02-0.12**	**0.009**
Gender	0.49	-0.43 to 1.4	0.30	0.66	-0.26 to 1.59	0.16
Ethnicity	-0.50	-1.12 to 0.13	0.12	-0.48	-1.12 to 0.16	0.14
eGFR	-0.009	-0.03 to 0.01	0.37	-0.01	-0.03 to 0.009	0.29
AER	-0.14	-0.42 to 0.14	0.40	-0.12	-0.40 to 0.17	0.42
**SBP**	**0.04**	**0.02 to 0.07**	**0.003**	N/A	N/A	N/A
MAP	N/A	N/A	N/A	0.03	-0.01 to 0.08	0.14
**sKlotho**	**-0.65**	**-1.24 to -0.06**	**0.03**	**-0.83**	**-1.42 to -0.24**	**0.006**
HbA1c	0.30	-0.02 to 0.63	0.07	0.27	-0.06 to 0.57	0.11
BMI	-0.001	-0.017 to 0.016	0.94	-0.003	-0.02 to 0.014	0.74
Calcium	-1.62	-5.93 to 2.69	0.46	-2.26	-6.66 to 2.14	0.31
Phosphate	-0.85	-2.67 to 0.96	0.36	-0.92	-2.79 to 0.93	0.33
AER, albumin excretion rate; Ao-PWV, aortic pulse wave velocity; eGFR, estimated glomerular filtration rate; MAP, mean arterial pressure; SBP, systolic blood pressure;BMI ,body mass index.						

Supplementary figure S1. Association of sKlotho with eGFR in 172 people with T2D and DKD

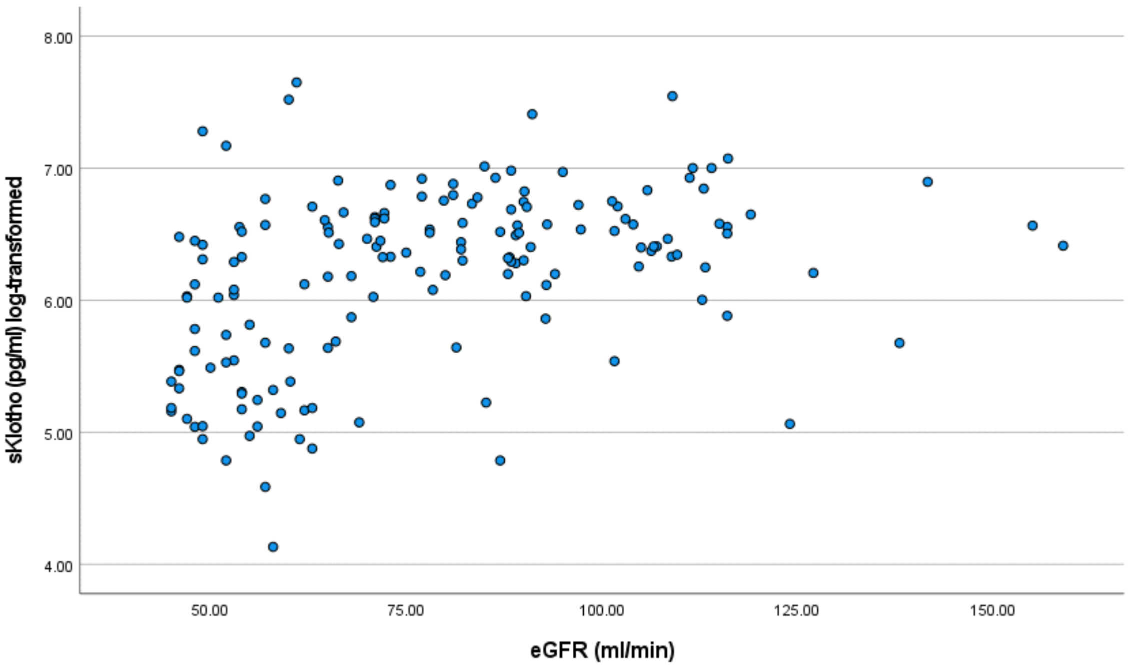

Supplementary figure S2. Soluble klotho attenuates AngII-stimulated $[Ca^{2+}]_i$ transients. HASMC were treated with soluble klotho (1 nM, 24 h) before loading with the fluorescent Ca2+ indicator Fura-2 AM (2 μM, 45 min). [Ca2+]i was monitored after stimulation (arrow) with AngII (100 nM) or Kreb's buffer alone (Vehicle). Mean [Ca2+]i response was expressed as fold change from baseline (first 30 sec of recording) from 4 independent HASMC cultures.

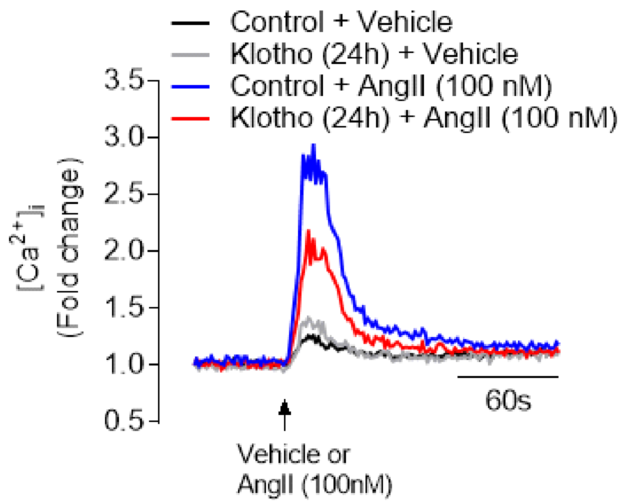

Supplement: Supplementary File (PDF) [file mmc1.pdf]
